# Effect of Monovalent Cations on the Structure and Dynamics of Multimodal Chromatographic Surfaces

**DOI:** 10.1021/acs.langmuir.3c03294

**Published:** 2024-03-22

**Authors:** Sabrina C. Lau, Camille L. Bilodeau

**Affiliations:** † Dublin High School, Dublin, California 94568, United States; ‡ Department of Chemical Engineering, University of Virginia, Charlottesville, Virginia 22903, United States

## Abstract

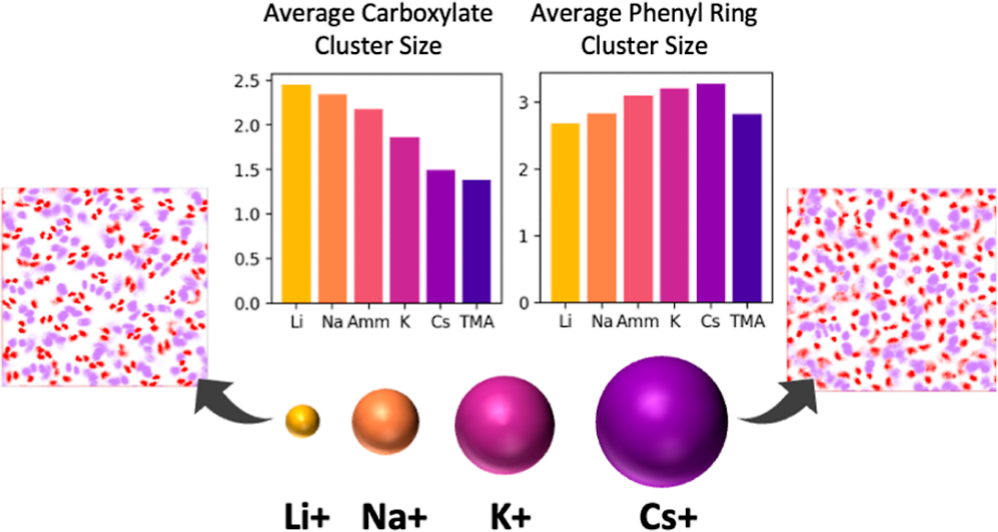

While multimodal (MM) chromatography is a promising approach for purifying proteins, the lack of a fundamental understanding of how ion–ligand interactions govern selectivity limits its use in the biopharmaceutical industry. This study uses molecular dynamics simulations to study the interactions between simple monovalent cations and two commonly used structurally similar multimodal chromatography ligands, the Capto ligand and Nuvia cPrime, immobilized on the surface. On the Capto ligand surface, ion presence and type play a key role in modulating the formation of phenyl rings and carboxylate clusters. The flexible linkage attaching the Capto ligand to the self-assembled monolayer (SAM) surface allowed multiple ligands to form interactions with the small cations, while large cations interacted less strongly, following the order Li^+^ > Na^+^ > K^+^ > Cs^+^. Thus, smaller cations resulted in greater ordering on the surface and lower ion diffusivities, while larger cations resulted in less ordering and higher ion diffusivities, following the order Li^+^ < Na^+^ < K^+^ < Cs^+^. In contrast, due to the rigid attachment of Nuvia cPrime to the SAM surfaces, the cations bound less strongly and had a much smaller effect on ligand clustering or ordering. Additionally, ions in the presence of the Nuvia cPrime surface had generally greater diffusivities than those in the presence of the Capto ligand. Overall, the interaction of cations with the multimodal ligands can lead to unique configurations on the SAM that likely contribute to differential behavior in biological separations.

## Introduction

Chromatography, the dominant method for protein purification in the biopharmaceutical industry, often separates proteins using changes in salt concentration to selectively disrupt protein–resin interactions. In recent years, multimodal (MM) chromatography has emerged as a powerful tool for achieving challenging protein separations within a single column.^1^ Unlike single-mode chromatography methods such as ion-exchange chromatography (IEX) or hydrophobic interaction chromatography (HIC), MM chromatography separates proteins by using ligands that are capable of multiple modes of interaction.^2^ Despite this, MM ligand–protein interactions have proven to be challenging to understand and predict, limiting the use of MM chromatography in industry.^1,3^ This is in part because, while ion–surface interactions have been well studied for simple charged and hydrophobic surfaces, they are poorly understood for multimodal surfaces. Therefore, to understand multimodal chromatography at the molecular level, it is necessary to characterize multimodal ligand–salt interactions in the context of a chromatography surface.

Many different experimental and computational techniques, such as NMR, AFM, and molecular dynamics (MD) simulations,^4−8^ have been used to study molecular-scale interactions in MM chromatography. Most of these studies have focused on characterizing interactions between the biological molecule and either the chromatography ligands or the ions. In contrast, how ions interact with multimodal chromatographic surfaces is not well understood. Our previous MD simulations suggested that the Capto ligand, a commonly used multimodal chromatography ligand, can interact strongly with sodium counterions when immobilized on a surface, coordinating them in geometries that are reminiscent of metal chelators.^8^

**Figure 1:**
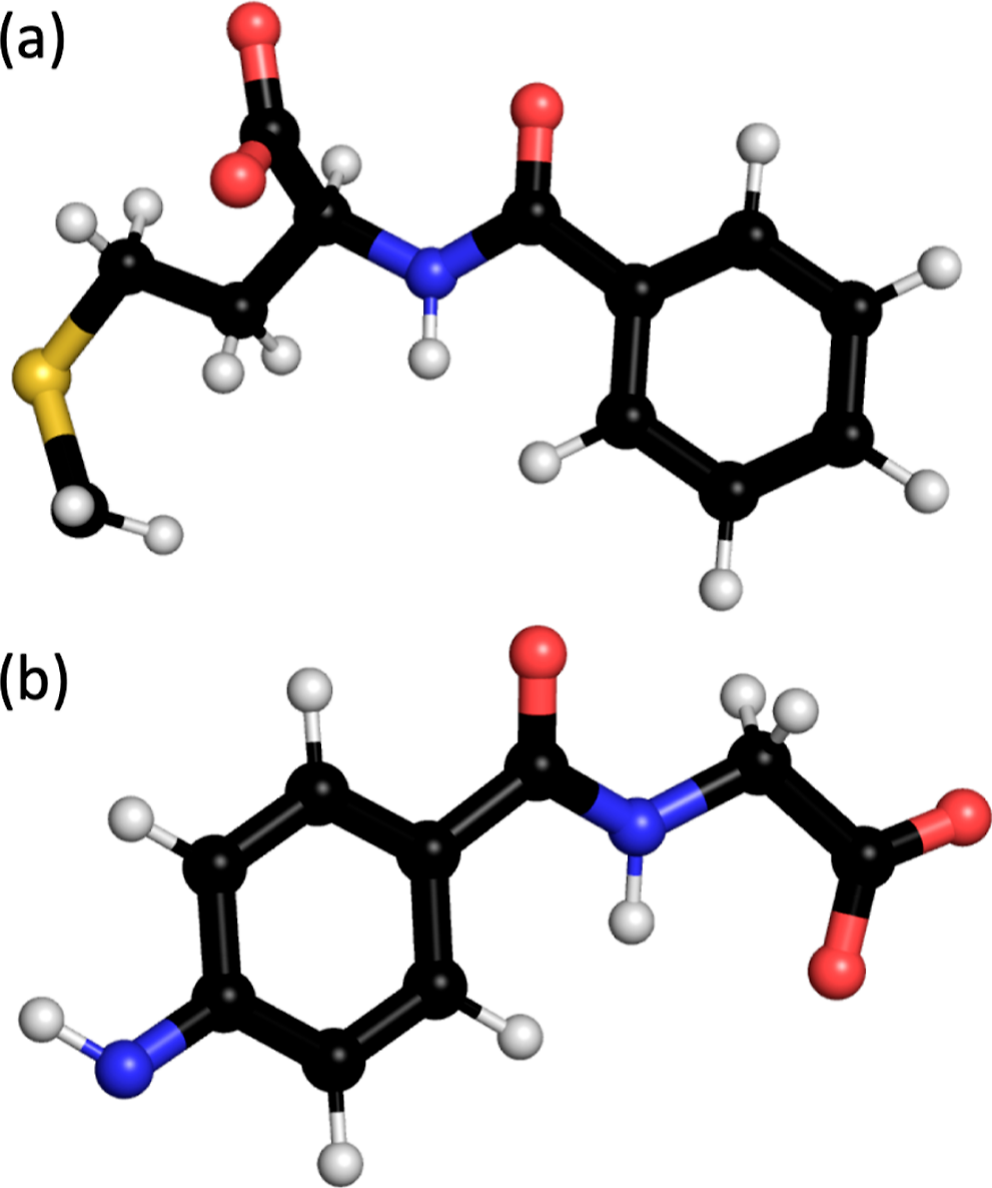
Ball and stick representation of the multimodal ligands of (a) Capto ligand and (b) Nuvia cPrime. The Capto ligand is attached to the SAM surface through the methylene group, while the Nuvia cPrime ligand attaches through the amine. Colors: carbon: black, hydrogen: white, oxygen: red, nitrogen: blue, and sulfur: yellow.

In this paper, we used molecular dynamics simulations to explore how ion type and ligand structure impact ion–ligand interactions in the context of ligand-functionalized surfaces. We focused on two commonly used, commercially available multimodal chromatography ligands, the Capto ligand and Nuvia cPrime (Figure 1), which exhibit different selectivities from one another despite being structurally similar.^9,10^ Specifically, molecular dynamics simulations were performed on a diverse panel of cations (Li^+^, Na^+^, K^+^, Cs^+^, NH_4_^+^, and tetramethylammonium) that have different charge densities and hydrogen bonding abilities with the MM ligands. Although these cations have the same charge, they are physically different and have differential behavior with not only the carboxylates but also the phenyl groups of the MM ligands, which likely has significant effects on their interactions with biologics and their ability for performing separations. We quantify the effect of the ion type on ligand–ligand interactions, ion placement, and dynamics within the multimodal surface.

## Materials and Methods

As was done in our previous work, simulations were performed with ligands immobilized at a constant ligand density of 1 ligand/nm^2^ on a self-assembled monolayer (SAM), which approximately corresponds to the commercially available density for the Capto ligand and Nuvia cPrime chromatography resins. Here, the Capto ligand corresponds to the commercially available Capto MMC ligand (produced by Cytiva) but differs in that it lacks the polyglycerol linkage to the resin. Nuvia cPrime corresponds to the commercially available resin by the same name (produced by Bio-Rad Laboratories). Details of the commercially available resins can be found in Supporting Information. Both the Capto ligand and Nuvia cPrime correspond to hippuric acid immobilized on the surface via alkyl thiol and amine groups, respectively. The SAM was created from alkyl thiol strands terminating in either hydroxyl groups (which lend the surface hydrophilicity to mimic the properties of a typical chromatographic matrix) or ligands. For strands terminating in ligands, each ligand was immobilized through connecting the base atom (Figure 1) to an alkyl thiol chain containing 10 carbons. The box size was 11.0 × 10.4 × 10.0 nm^3^, and each simulation contained 528 alkyl thiol SAM strands, with 132 strands terminating in a ligand and the remaining strands terminating in a hydroxyl group. The ligands were placed evenly across the surface in a hexagonal array. A harmonic potential of 1000 kcal/mol·Å2 was used to restrain the sulfur atom and the seventh carbon from the sulfur to maintain the structure of the surface, as was done in previous studies. While we expect that this is an idealized representation of ligand arrangement on the surface, we note that the ligands are likely evenly spaced in the real chromatographic resin due to steric interactions upon the ligand-surface conjugation. For greater details on the configuration setup, we refer the reader to our previous publication.^8^

Classical molecular dynamics simulations were performed using the GPU accelerated program pmemd.cuda in Amber (Version 20).^11,12^ The GAFF force field was used to model the surface with both multimodal ligands, the Capto ligand, and Nuvia cPrime.^13^ The surfaces were solvated with TIP3P water,^14^ and 132 cations were added to neutralize the system. The alkali metal cations (Li^+^, Na^+^, K^+^, and Cs^+^) were modeled using the parameters of Joung and Cheatham.^15^ The ammonium (NH_4_^+^) and tetramethylammonium (TMA) ions were modeled utilizing the parameters of Heyda et al.^16^ In order to allow for electroneutrality, simulations without cations were performed in the presence of a neutralizing plasma, which has the effect of introducing a diffuse background neutralizing charge, as has been done previously.^17^ RESP charges were used to assign partial charges to the Capto ligand and Nuvia cPrime ligand.^18^ The density of the water was simulated to be 1.0 g/mL. The energy of the system was minimized using a combination of steepest descents and conjugate gradients before dynamics. The molecular dynamics simulations were performed in an *NP*γ*T* ensemble using the Langevin integrator with a collision frequency of 3 ps^–1^.^19^ The simulations were performed utilizing a constant surface tension of 10 dyn/cm along the *XY* plane (semi-isotropic), while the *Z*-direction can change independently.^20^ The system was coupled to a Monte Carlo thermostat at 300 K. Nonbonded interactions were cutoff at 9 Å. The electrostatics was treated using particle mesh Ewald summation with a 9 Å real space cutoff and a 1 Å grid.^21^ SHAKE was used to constrain bonds containing hydrogens.^22^ A 2.0 fs time step was used, and each simulation was run at 50 ns. Four replicate simulations for each system were performed to better sample the conformations.

The molecular clustering was performed using the program OVITO^23^ using a distance cutoff of 4 Å between any of the carbons of neighboring phenyl groups over the last 20 ns of dynamics. A 5 Å cutoff was used for any atom of the carboxylate (carbon or oxygen) with its neighbor.

## Results and Discussion

### Ion Effect on the Phenyl Ring and Carboxylate Cluster Formation

In previous simulation studies, we found that the geometric arrangement of the phenyl ring and carboxylate groups played a large role in determining how ligands interact with one another on a surface. Specifically, we found that when a phenyl ring is immobilized on a surface via a flexible linker, it aggregates with neighboring ligands to form clusters (observed for the Capto ligand). We also observed that, in the presence of Na^+^ counterions, carboxylate groups of neighboring Capto ligands showed a tendency to cluster together. We expect that the size and distribution of these hydrophobic and charged clusters will affect how the chromatography surface interacts with proteins with different surface properties, altering the chromatographic retention behaviors. This led to the following questions: how are phenyl ring and carboxylate clusters affected by the presence of counterions? Further, is it possible to tune cluster formation and surface pattern formation by altering the type and size of the counterion?

To explore these questions, we performed MD simulations of the Capto ligand immobilized at a standard ligand density (1 ligand/nm^2^) on an alkyl thiol SAM surface, where each alkyl chain was terminated in a hydroxyl headgroup. Simulations were first performed in the absence of any ions and then in the presence of a series of ions of increasing size: Li^+^, Na^+^, NH_4_^+^, K^+^, Cs^+^, and TMA. We note that NH_4_^+^ and K^+^ are similar in their size but differ in that NH_4_^+^ can form hydrogen bonds, while K^+^ cannot, which causes differences in the coordination number and geometry in their first hydration sphere.^24^ In all of the MD simulations, almost all of the cations were located near the SAM surface, as has been observed in other simulations of charged SAM surfaces.^25,26^ This is because the high electrostatic charge created by the local concentration of carboxylates in the Capto ligands or Nuvia cPrime attracts all of the cations to congregate on the surface.

**Figure 2:**
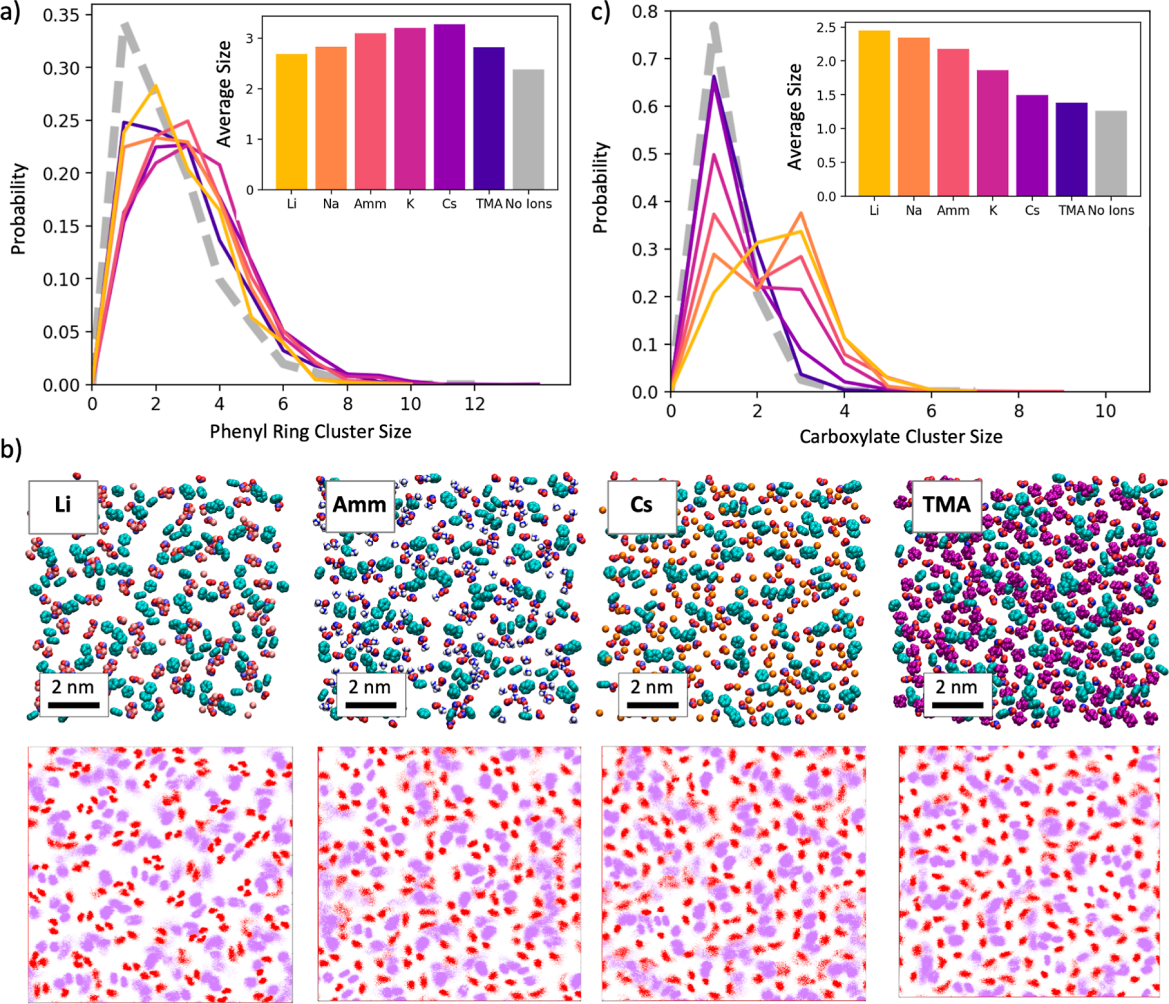
(a) Probability distribution of observing a phenyl ring cluster of a given size. Gray dashed lines refer to the simulations without counterions, and colored solid lines refer to the simulations with counterions. Inset: average phenyl ring cluster size for simulations containing different ions. (b) Top: snapshots from simulations containing lithium (far left), ammonium (middle left), cesium (middle right), and TMA (far right). Bottom: atomic density distributions of the phenyl rings (purple) and carboxylate groups (red) in the plan of the surface. (c) Probability distribution of observing a carboxylate cluster of a given size.

Figure 2a illustrates the probability distribution for the phenyl ring cluster size as a function of ion type. In the absence of counterions, the phenyl rings formed smaller and fewer clusters than were observed in any of the ion-containing simulations, with an average cluster size of 2.4 phenyl rings (compared with 3.3 for simulations containing Cs^+^). This illustrates that the phenyl ring cluster formation previously observed for multimodal surfaces is ion-mediated, with counterions increasing cluster formation regardless of their size.

Ions with larger radii were generally found to increase phenyl ring cluster formation more than smaller ions, following the trend Li^+^ < Na^+^ < K^+^ < Cs^+^. This is consistent with previous simulation and experimental studies that have shown that larger ions with lower charge densities can interact more favorably with hydrophobic molecules,^27,28^ promoting ligand–ligand association. Surprisingly, the phenyl rings formed fewer clusters in the presence of TMA, despite TMA being significantly larger than any of the other ions. We hypothesize that the large size of TMA causes it to crowd out the phenyl rings instead of promoting aggregate formation. This suggests that in the context of a surface, there exists an upper bound to the ion size that can effectively promote phenyl ring cluster formation, beyond which ions compete with phenyl–phenyl interactions. This effect can be observed in Figure 2b (far right), which illustrates the size and coverage of TMA on the ligand-functionalized surface.

Figure 2c illustrates the probability distribution for the carboxylate cluster size as a function of the ion type. In the absence of counterions, carboxylate cluster formation was dramatically reduced, with an average cluster size of only 1.26 (compared with 2.45 for simulations containing Li^+^). This is consistent with the hypothesis that carboxylate cluster formation is driven by the association of multiple carboxylate groups with cations.

**Figure 3:**
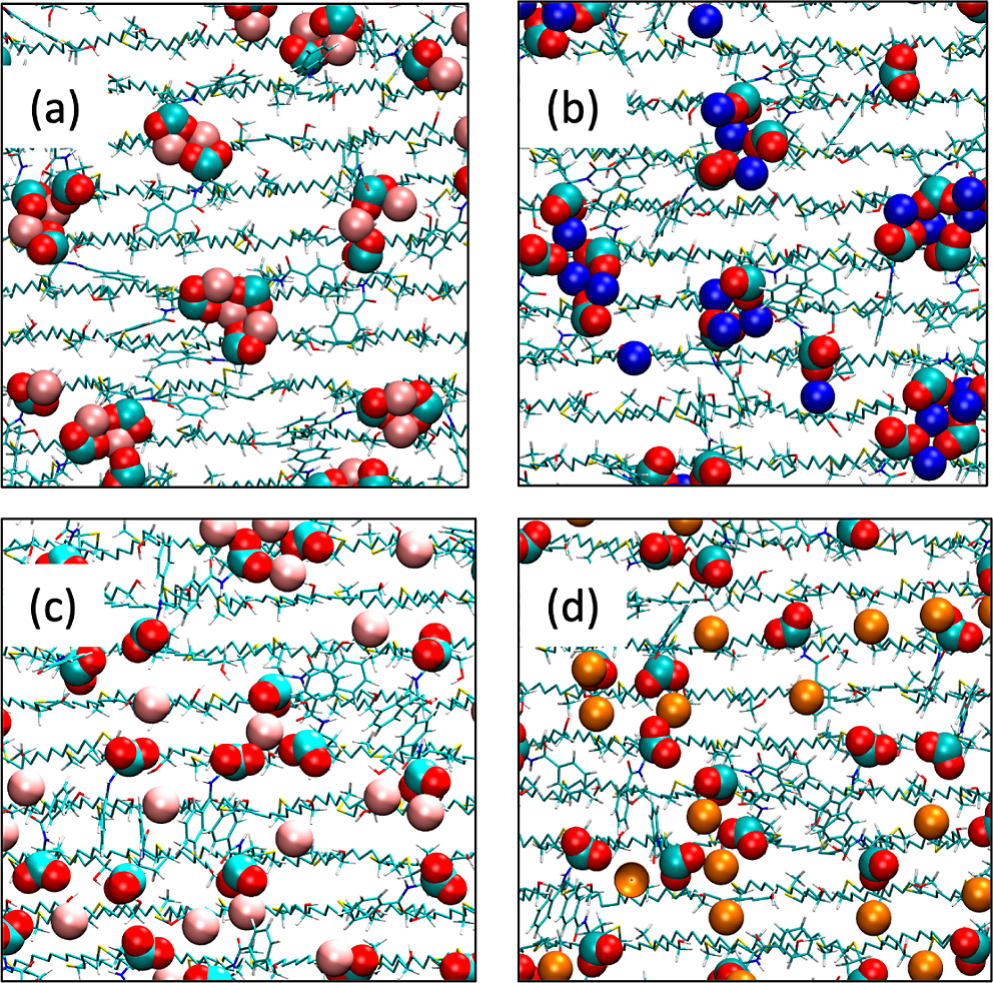
Snapshots illustrating clustering between carboxylates of the Capto ligand and cations (a) lithium, (b) sodium, (c) potassium, and (d) cesium. Colors: carbon: cyan, oxygen: red, lithium: dark pink, sodium: blue, potassium: light pink, and cesium: orange.

In contrast to phenyl ring cluster formation, ions with smaller radii were found to promote carboxylate cluster formation, with cluster size following a reverse Hofmeister series Li^+^ > Na^+^ > K^+^ > Cs^+^.^29^ While this ordering is consistent with previously reported activity coefficients of alkali metal cations-acetate solutions,^30^ cation-carboxylate contact formation for the Capto ligand differs from that in solution. In water, a single monovalent cation tends to interact with a single acetate as a contact ion pair. In contrast, we observed that smaller cations formed multi-ion clusters that resembled structures present in the solid state, where multiple interactions stabilize and give long-range order to the structure.^31,32^ This type of ion clustering with long-range ordering has been seen for Li^+^ interacting with trifluoroacetate from all-atom MD simulations and is consistent with X-ray scattering experiments.^33^ The X-ray scattering experiments showed several sodium peaks at regular intervals that could be explained by sodium ions forming salt bridges between the carboxylate oxygens. Figure 2b illustrates this phenomenon, with Li^+^ ions forming tight, multi-ion clusters, NH_4_^+^ ions forming looser clusters, and Cs^+^ ions remaining largely unbound from the carboxylates. While simulations containing Li^+^ counterions formed tight clusters containing 2–3 carboxylates (Figure 3a), these clusters were less ordered for simulations containing cations of increasing size (Figure 3). Interestingly, although NH_4_^+^ is similar to K^+^ in size, it had a larger impact on carboxylate cluster formation. We hypothesize that this can be attributed to NH_4_^+^-carboxylate hydrogen bonding, which allows NH_4_^+^ to interact more strongly than the equivalent monovalent cation. This is consistent with previous first-principle molecular dynamics simulations of K^+^ and NH_4_^+^, which have shown that the first hydration sphere of NH_4_^+^ is much more tightly packed and ordered than K^+^ due to its hydrogen bonding ability.^24^

In contrast, the different cations had a minimal effect on the interaction between the phenyl and carboxylate groups in Nuvia cPrime simulations. The Nuvia cPrime ligand is coordinated to the SAM surface through the amine to form a rigid connection, where the phenyl group is located closer to the surface and the carboxylate is directed into the solvent. Although the cations congregate on the Nuvia cPrime surface, they tend to form one-to-one interactions between the cation and carboxylate and rarely form multiple interactions with the carboxylates. The rigidity of the Nuvia cPrime bond to the SAM surface does not allow direct interaction between the phenyl groups.^8^

### Strength of Ion–Surface Interactions

The strength of ion–ligand interactions plays an important role in governing selectivity and retention time in multimodal chromatographic systems. Specifically, when a protein binds to a chromatographic surface, the counterion–ligand interactions must be disrupted in order to allow the protein to replace the ion on the surface. To quantify the overall strength of ion–surface interactions, the probability of the ion being in the bound layer versus the bulk was calculated as
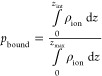
1
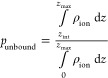
2where *z*_int_ refers to the location of the edge of the bound layer, defined as the *z* coordinate at which ligand density reaches zero, and where *z*_max_ refers to the length of the simulation box in the *z* direction.

**Figure 4:**
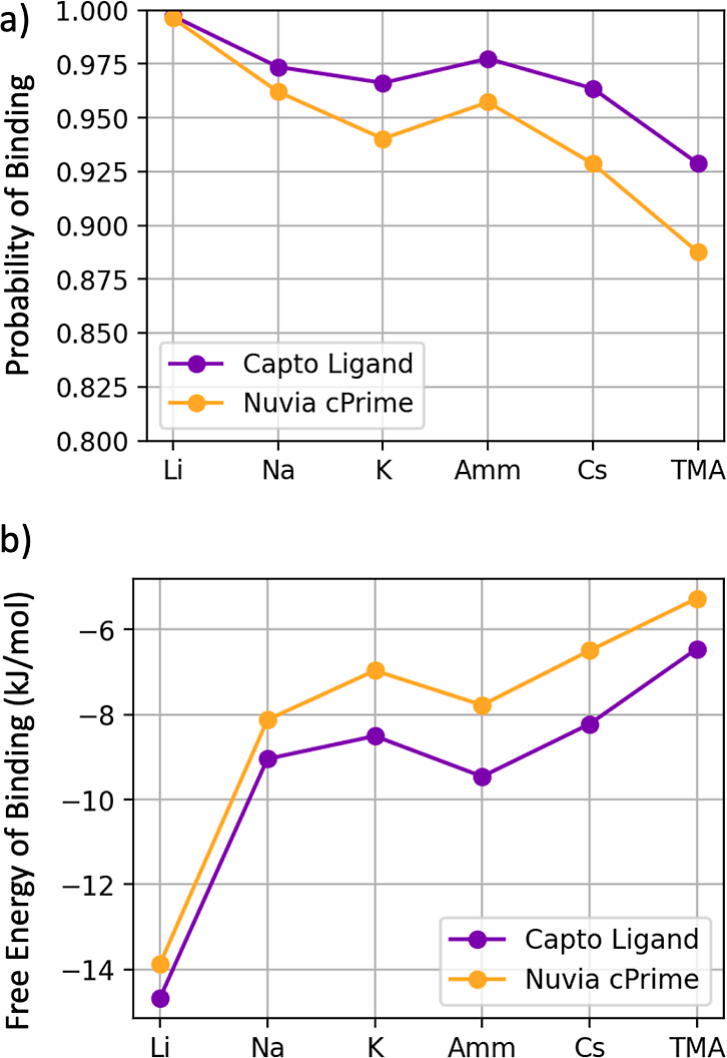
(a) Probability of and (b) free energy of each ion binding to the Capto ligand and Nuvia cPrime surfaces.

Figure 4a illustrates *p*_bound_, and Figure 4b illustrates the free energy of moving from the bulk into the bound layer, Δ*G*_binding_, which can be calculated as −*k*_B_*T* ln *p*_bound_/*p*_unbound_. Overall, smaller ions were found to bind to both surfaces more strongly than larger ions. One exception to this trend was NH_4_^+^, which, despite being the same size as K^+^, bound more strongly to both ligands due to its ability to form hydrogen bonds with the carboxylates. Additionally, we found that all ions studied bound more strongly to the Capto ligand surface than to the Nuvia cPrime surface, although for all ions the magnitude of this difference was less than 1 *k*_b_*T* (2.48 kJ/mol), indicating that this difference is less than the magnitude of thermal fluctuations. We expect, based on previously developed ion exchange isotherms, that a lower (more favorable) free energy of binding for a given salt/resin combination will correspond to lower elution salt concentrations for proteins.

### Ion Ordering in the Bound Layer

**Figure 5:**
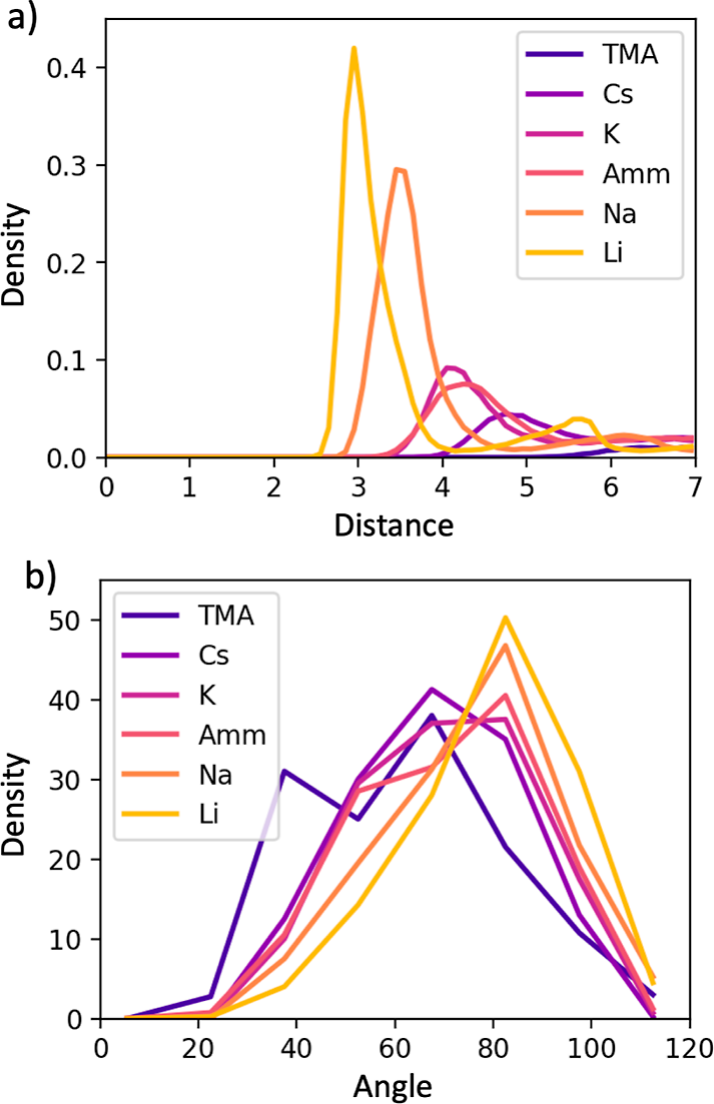
(a) Ion–ion radial distribution functions. (b) Angular distributions between the vectors formed by the carboxylic acid group and alkyl chain on the Capto ligand. The distance is given in Å. Density is in units of atoms/Å^3^, and angle is in degrees.

In the first section, we observed large carboxylate clusters on the Capto ligand surface that resembled structures present in the solid state for small ion sizes. To further quantify the ordering of the ions in the bound layer, Figure 5a illustrates the ion–ion density distribution, and Figure 5b illustrates the distribution of the angle formed by the ion, carboxylate oxygen, and carboxylate carbon. Li^+^ was found to exhibit tight, ordered clusters, with the ion–ion distribution containing a high/narrow nearest neighbor peak and a secondary peak observed further out corresponding to the nearest cluster (Figure 5a). The coordinating geometries of the carboxylate oxygens around the Li^+^ tended to be in-plane and, in some cases, form a square planar geometry around the cation (Figure 5b). This is consistent with the observed hydration structure around Li^+^. The smaller radii allow for 4 oxygens from the surrounding waters to coordinate the ion.^34,35^ Sodium exhibited slightly looser, less ordered clusters, with the ion–ion distribution containing a peak a bit further out, slightly broader, and a significantly more diffuse second peak (Figure 5a). The coordinating geometry of the carboxylate oxygens around Na^+^ was less rigid than that seen for Li^+^, with more oxygens deviating from the plane (Figure 5b). For the K^+^, NH_4_^+^, and Cs^+^ ion–ion distributions, this first peak is much more diffuse and the second peak is not visible (Figure 5a), and the carboxylate-ion angular distribution is increasingly broad. TMA is the only counterion for which no ordering was observed.

### Density Distribution of Bound Ions along the Surface Normal

**Figure 6:**
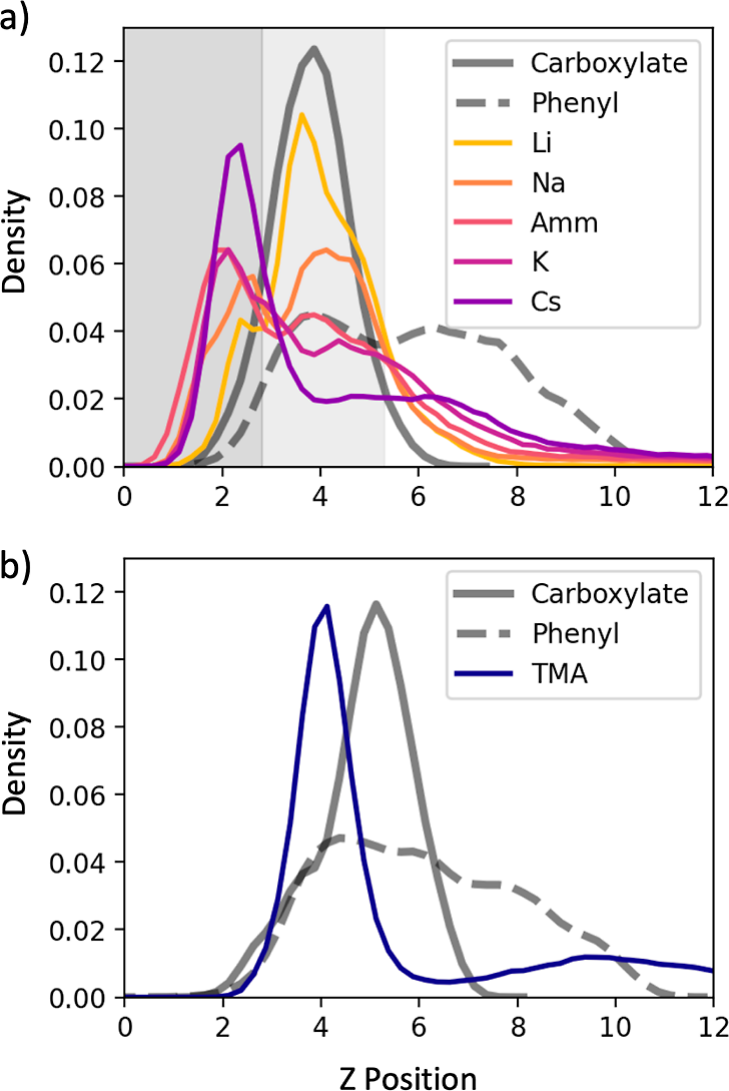
(a) Colored lines: ion density distributions were normal to the plane of the surface. Gray lines (solid and dashed): atomic density distributions are normal to the plane of the surface for different chemical moieties of the Capto ligand. Distributions are split into three regions: the left region (dark gray) indicates the region adjacent to the hydrophilic surface, the middle region (light gray) indicates the region in the same plane as the carboxylic acid groups, and the right region (white) indicates the outer region containing the phenyl rings. The boundary between the left region and the middle region is located at 3.2 Å, and the boundary between the middle and right regions is located at 5.7 Å. Boundary locations were determined based on inflections in the carboxylate density distribution. (b) TMA ion density (blue) and atomic density distributions for the different chemical moieties of the Capto ligand. TMA density is presented separately because TMA affects the atomic density distributions of the Capto ligand moieties. Distances are given in units of Å. Densities are in units of atoms/Å^3^.

In addition to studying the effect of cations on cluster formation on the Capto ligand surface, we were interested in understanding where ions accumulate in the simulation and why. To explore this, Figure 6a illustrates the ion density distribution along the *z* axis (the surface normal), broken up into three regions based on the carboxylate and phenyl ring densities. The first region (left) corresponds to the space directly adjacent to the hydroxyl-capped SAM surface. The second region (middle) corresponds to the space occupied by the carboxylate groups partially and by the phenyl rings, to a lesser degree. The third region (right) corresponds to the diffuse outer layer that was partially occupied by the phenyl rings. In all of the Capto ligand simulations except the simulation containing TMA counterions, the distributions of the phenyl ring, carboxylate, and surface densities were not affected by the identity of the cation (illustrated in Supporting Information).

The ion density distribution in the *z* dimension can be considered a balance among ion–surface interactions, ion-carboxylate interactions, and ion-phenyl ring interactions. Unsurprisingly, the Li^+^ density formed a narrow peak in the middle region, consistent with the fact that it remained primarily bound to the carboxylate clusters. As the cation size increased, the density was found to shift into the first and third regions, with Cs^+^ exhibiting a sharp peak near the hydroxyl-capped surface and a diffuse shoulder near the phenyl ring density. This shift can be attributed to a transition from a regime dominated by carboxylate-ion interactions to one dominated by surface/phenyl–ion interactions. This observation is consistent with previous simulation studies by Schwierz and co-workers, which have shown that ion-hydrophilic surface interactions increase with increasing ion size.^36^ Recently, a study of the adsorption of sodium dodecanoate at the air–water interface by Nguyen et al., using surface tension measurements, SFG spectroscopy, and MD simulations, showed that when the surfactant acetate headgroup is charged, Li^+^ binds strongly to the acetate, but when the headgroup is neutralized, Cs^+^ has stronger interactions.^28^

Figure 6b illustrates the density distribution of the TMA ions in the *z* dimension. Similar to the other large ions, TMA exhibited a sharp peak near the hydroxyl-capped surface, indicating strong ion–surface interactions. The bulkiness of the TMA counterions, however, caused them to push the carboxylate and phenyl ring groups away from the surface, consistent with the crowding-out effect described in the previous section.

**Figure 7:**
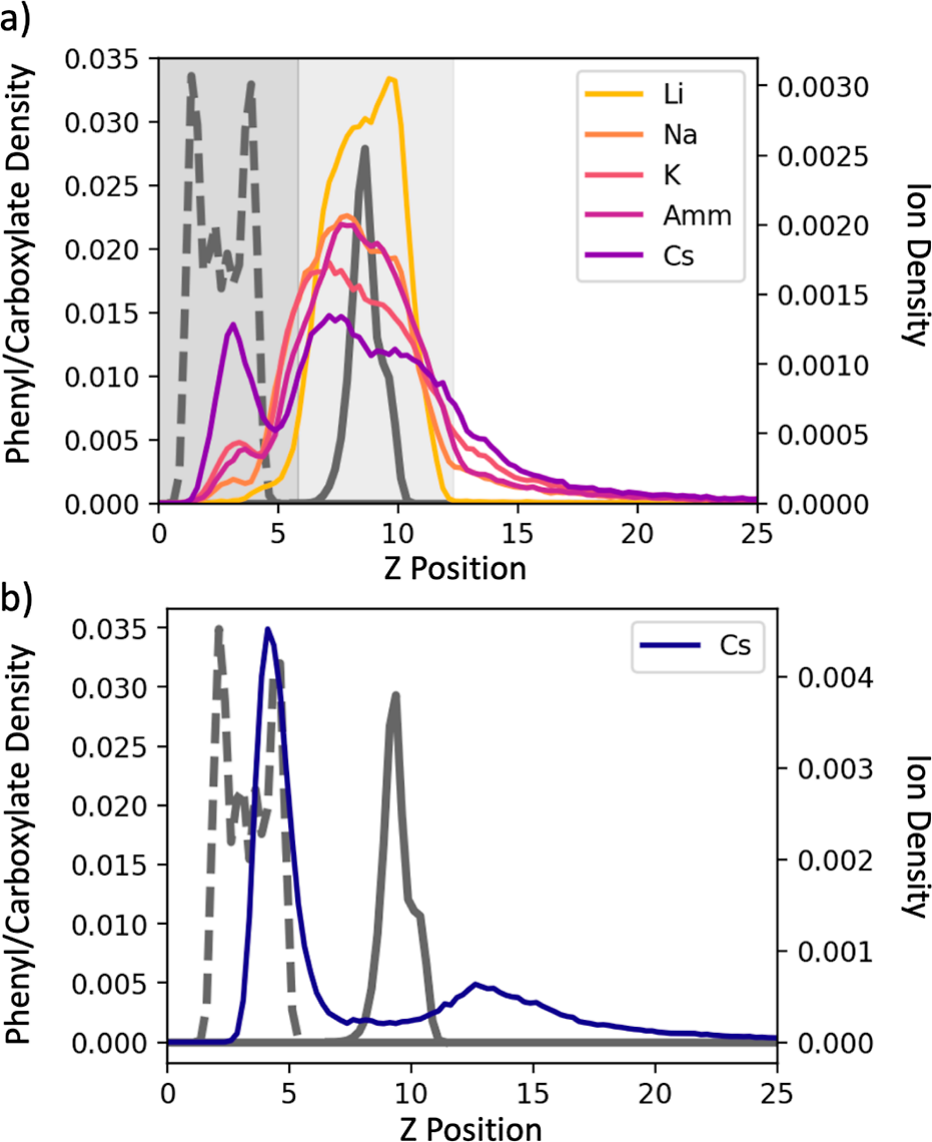
(a) Colored lines: ion density distributions are normal to the plane of the Nuvia cPrime surface. Gray lines: atomic density distributions normal to the plane of the surface for the Nuvia cPrime phenyl group (dashed) and carboxylate group (solid). Distributions are split into three regions: the left region (dark gray) indicates the region adjacent to the hydrophilic surface and in the plane of the phenyl groups, the middle region (light gray) indicates the region in the same plane as the carboxylate groups, and the right region (white) indicates the outer region. The boundary between the left region and the middle region is located at 5.9 Å, and the boundary between the middle and right region is located at 12.7 Å. Because the density distribution for the carboxylate groups is far narrower for Nuvia cPrime than for the Capto ligand, boundary locations were determined in order to fully encompass the carboxylate density distribution. (b) TMA ion density (blue) and atomic density distributions for the different chemical moieties of Nuvia cPrime. TMA density is presented separately because TMA affects the atomic density distributions of the Capto ligand moieties. Distances are in units of Å. Density is in units of atoms/Å^3^.

**Figure 8:**
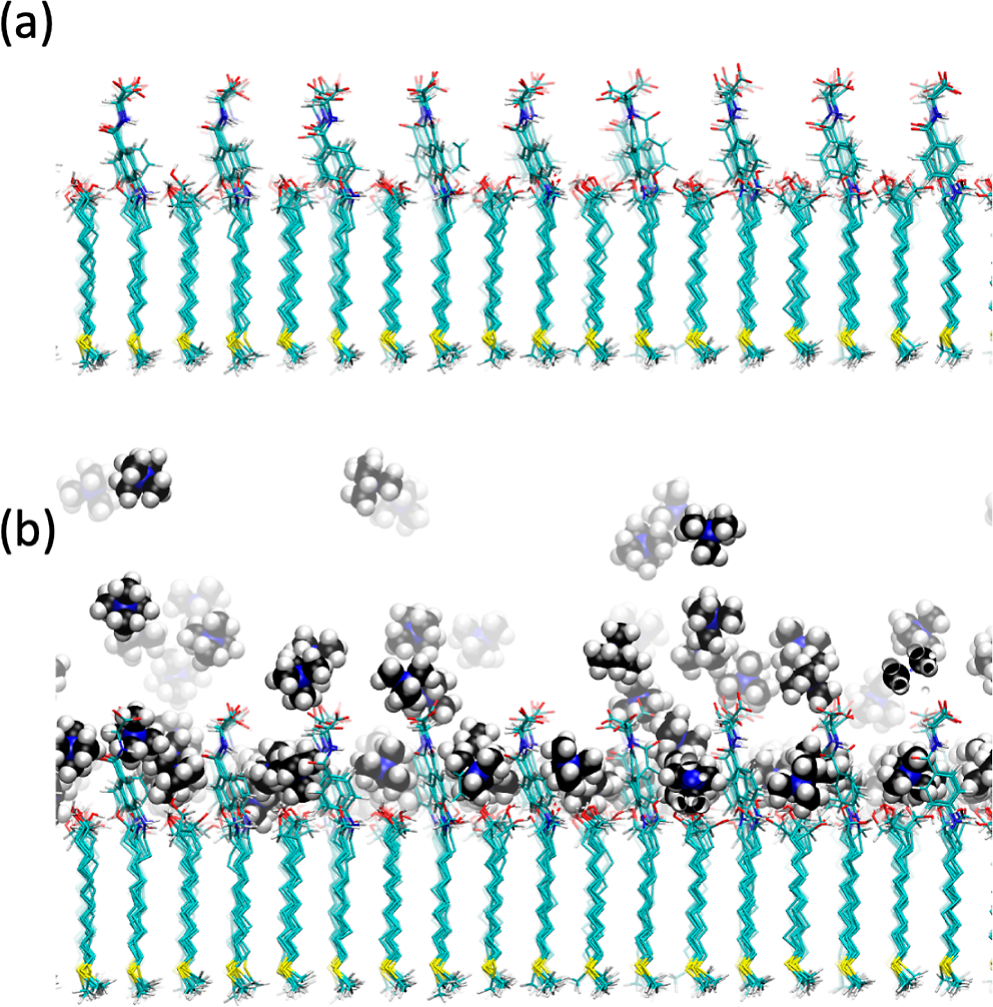
(a) Snapshot of the Nuvia cPrime surface. The rigid ligand attachment to the SAM allows for the formation of deep canals on the surface. (b) Snapshot showing the inclusion of the TMA ions on the Nuvia cPrime surface. The TMA preferentially interacts with the phenyl group rather than the carboxylates.

As shown in Figure 7, the overall behaviors of the ions near the Nuvia cPrime surface are similar, with smaller ions concentrating near the carboxylate density (middle region, shown in light gray) and larger ions shifting toward the phenyl ring density and the surface below (left region, shown in dark gray). Interestingly, this shift in density toward the surface for Cs^+^ appears to be less pronounced near the Nuvia cPrime surface (Figure 7a, purple), while the shift for TMA (Figure 7b) is more pronounced. This difference is because of the rigid connection of Nuvia cPrime to the SAM surface, which creates canals along the surface that are able to accommodate the bulky, hydrophobic TMA cations (Figure 8). We note that it is likely the number of alkali metal cations, except Li^+^, in the canals is underestimated since classical force fields do not explicitly take into account cation–π interactions.^37^

### Dynamics of Ions in the Bound Layer

**Figure 9:**
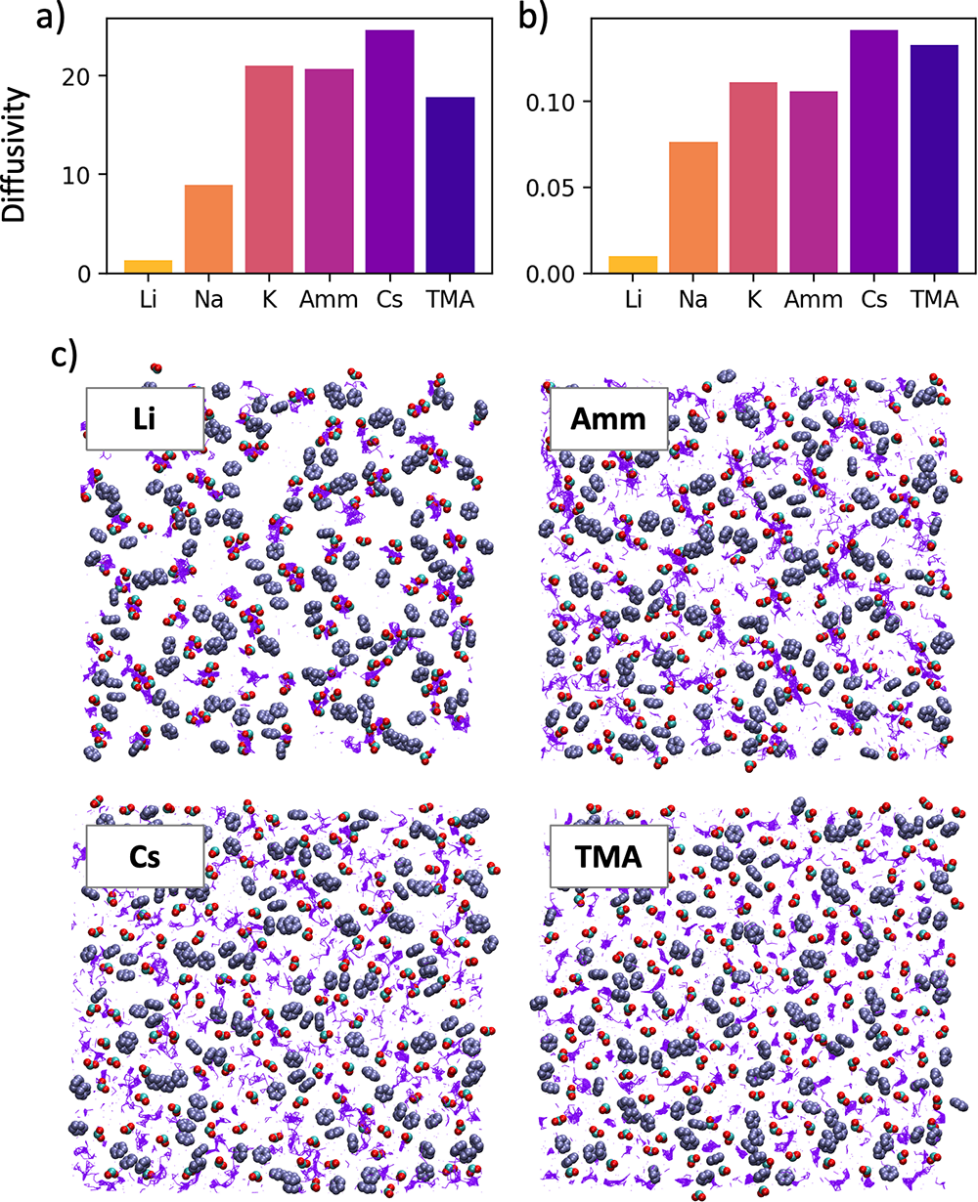
(a) Diffusivity of ions in the bound layer (×10^–9^ m^2^/s). (b) Diffusivity of ions in the bound layer normalized by their diffusivities in bulk water. (c) Ion path shown over a 20 ns production run, shown with purple lines. For reference, the lines are overlaid with a snapshot of the phenyl rings and carboxylic acid groups on the surface. Phenyl ring carbons are shown as purple, carboxylic acid carbons as cyan, and carboxylic oxygens as red (all ligand atoms are shown in space-fill).

In addition to studying equilibrium ion–surface interactions, we also explored dynamics by calculating the diffusivity of the ions in the bound layer on the Capto ligand surface. Consistent with the previous picture, where the quantity and ordering of ion-carboxylate interactions increased with decreasing size, we found that smaller ions diffused far more slowly than large ions, with diffusivity following the trend Li^+^ < Na^+^ < K^+^ < Cs^+^ (Figure 9a). We found that TMA had a lower diffusivity than Cs^+^,^38,39^ which we attribute to the fact that TMA has a lower diffusivity in bulk water. To illustrate this, Figure 9b shows that ion diffusivity in the bound layer normalized by diffusivity in the bulk follows the trend Li^+^ < Na^+^ < K^+^ < Cs^+^ < TMA.

To illustrate ion mobility in the plane of the surface, Figure 9c shows the ion path as a purple line over the course of the 20 ns production run. Li^+^ ions were largely observed to move slightly within a single cluster for the duration of the simulation. Cs^+^ and TMA ions were found to move randomly among the ligands. In contrast, the NH_4_^+^ trajectory formed lines between the carboxylates, appearing to move back and forth between larger groups of carboxylates over the course of the trajectory. We hypothesize that this phenomenon is the result of directional hydrogen bonding, allowing the NH_4_^+^ to interact with carboxylates to form a “zipper-like” motif.

**Figure 10:**
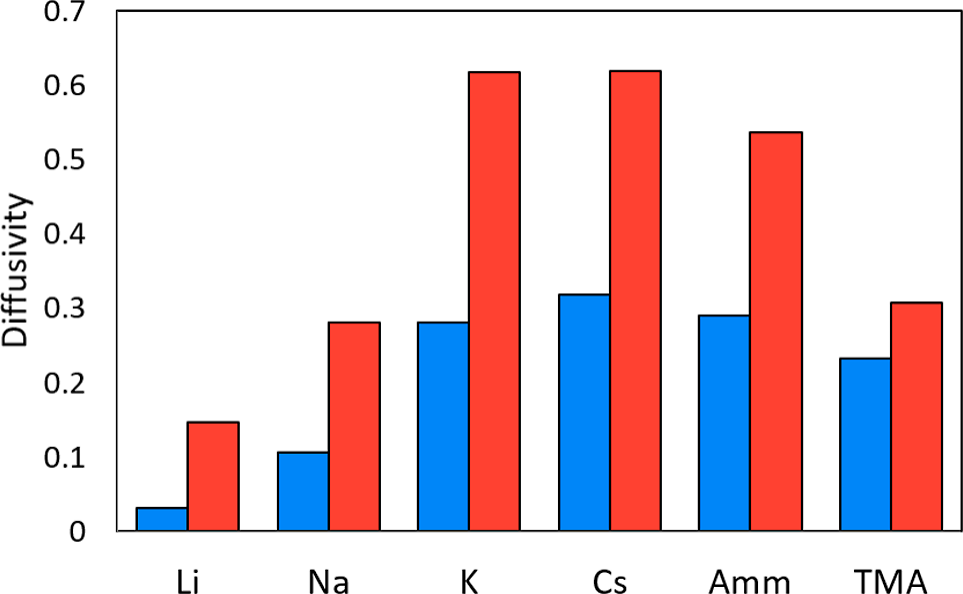
Comparison of the diffusivity of cations in the Capto ligand (blue) and Nuvia cPrime (red) surfaces (×10^–9^ m^2^/s).

Interestingly, the flexibility of the MM ligands also affected the diffusivity of the cations. In the simulations of the Nuvia cPrime surfaces, each of the ions diffused more rapidly than in the Capto ligand simulations, even though the ligands have the same charge (Figure 10). The ability of the Capto ligand to form stronger interactions via multiple contacts with the counterion significantly hindered their diffusion. The diffusion of the cations in the Nuvia cPrime simulations was, in most cases, double that of the analogous Capto ligand simulations. The diffusion coefficient of TMA was much closer between the two SAM surfaces. This is likely because TMA did not interact strongly with the carboxylates, so increased carboxylate flexibility had a reduced effect. However, even with TMA being able to reside in the canals formed by the Nuvia cPrime ligands, the diffusion was still more rapid than with the Capto ligands.

## Conclusions

Multimodal ligands have great promise in separating biologics through their ability to interact with molecules through hydrogen bonding, electrostatics, and the hydrophobic effect. Most theoretical descriptions of the effect of salt on protein retention focus on changes in dielectric or salting in/salting out effects. Here, we show that, in addition to these descriptions, ions and ligands can influence each other via a number of other mechanisms. Small cations such as Li^+^ or Na^+^ were found to interact strongly with the carboxylates of the Capto ligand due to their high charge density and flexibility, while larger cations were associated more weakly with the SAM. A similar behavior has been seen previously with carboxylate-terminated SAM surfaces,^25,36^ as well as with the carboxylates of methacrylic acid,^40^ and from X-ray absorption spectroscopy.^41^ This behavior of the cations with acetate is dictated by not only the anion’s charge. Surface tension experiments and SFG spectroscopy show that large cations have a greater preference for the anionic headgroups of sodium dodecyl sulfate (SDS),^42^ which has the opposite behavior to sodium dodecanote (SL).^28^ This trend is also seen for micelle-to-vesicle transitions of solutions SL and dodecyltrimethylammonium bromide (DTAB), which are strongly influenced by Li^+^ and Na^+^, but K^+^ and Cs^+^ have minimal affects.^43^ The opposite trend occurs for solutions of SDS/DTAB.^44^ The added anions did not influence the vesicle transition.

The reversal of the Hofmeister series in the presence of carboxylates of the Capto ligands can be explained by the concept of matching water affinities, as acetate is a strongly hydrated anion^30,45^ and prefers cations with high charge density (Li^+^ and Na^+^).^46^ These strongly hydrated ion pairs can form stable contact ion pairs in solution. The sulfate headgroup is more weakly solvated with a flexible hydration layer and prefers interactions with larger, less solvated cations. Weakly hydrated ions tend to stay away from strongly hydrated ions. Large cations were found to interact less strongly with the carboxylates but more strongly with the phenyl groups of the Capto ligand surface, creating large hydrophobic patches on the SAM surface. Additionally, the ion type was found to significantly impact the ion location within the SAM surface, with smaller ions concentrating in the plane of carboxylates and larger ions concentrating beneath the ligand density close to the hydroxyl surface. Finally, the ion type and the ligand structure were found to significantly impact ion diffusivity, with ions diffusing twice as fast near the Nuvia cPrime surface compared with those near the Capto ligand surface.

These ion–ligand interactions are likely to play a significant role in protein–surface binding in multimodal chromatographic systems. First, based on previous studies of patterned surfaces,^8,47^ it is expected that the size and distribution of phenyl ring clusters will impact the overall hydrophobicity of the surface as well as the orientational preferences of protein surface interactions. Therefore, we expect that the changes in the phenyl ring cluster distribution may lead to changes in the apparent hydrophobicity or selectivity. Additionally, we expect that for ions that bind more strongly, the energetic barrier required to displace these ions will be larger, changing protein binding energetics. Finally, ion diffusivity was higher on the Nuvia cPrime surface than on the Capto ligand surface, suggesting that the kinetics of displacing these ions may be faster. In the future, it would be valuable to explore the effect of different ion types on the strength and kinetics of protein-binding interactions. Further, given that proteins are often bound and eluted at different salt concentrations, it would be valuable to explore how these effects change with changes in ion concentration.

## Supporting Information Available

Distributions of carboxylate and phenyl ring densities as a function of distance in the *z* dimension and an illustration of the definition of the carboxylate and alkyl vectors (PDF)
