# Supplementary material for: Effect of Monovalent Cations on the Structure and Dynamics of Multimodal Chromatographic Surfaces
Source: Langmuir. 2024 Mar 22;40(13):6694–702. doi: 10.1021/acs.langmuir.3c03294 (PMC10993413; doi:10.1021/acs.langmuir.3c03294)
Supplement: Supplementary file 1 — la3c03294_si_001.pdf [file la3c03294_si_001.pdf]

The effect of monovalent cations on the structure and dynamics of multimodal chromatographic surfaces: Supplementary Information

Sabrina C. Lau[†] and Camille L. Bilodeau[*,‡]

†*Dublin High School, Dublin, CA 94568, United States*

‡*Department of Chemical Engineering, University of Virginia, Charlottesville, VA 22903, United States*

¶*3 pages, 2 figures, 0 tables*

E-mail: cur5wz@virginia.edu

Contents

1 Information about Commerically Available Resins **2**

2 Effect of concentration and co-ions on ion distribution **2**

3 SAM Surface Order Parameters **2**

1 Information about Commerically Available Resins

Capto MMC is a multimodal cation exchange resin sold by Cytiva Life Sciences that interacts with molecules via ionic, hydrophobic, and hydrogen bonding interactions. Capto MMC consists of the ligand shown in Figure 1 linked to an agarose base matrix by a polyglycerol linkage. Nuvia cPrime is also a mixed-mode cation exchange resin sold by Bio-Rad Laboratories. Nuvia cPrime consists of the ligand shown in Figure 1 linked to a hydrophilic polymeric matrix.

2 Effect of concentration and co-ions on ion distribution

Molecular dynamics simulations with of the CaptoMMC systems with 132 Na+ and a background NaCl concentrations of 0.2 M and 1 M show the additional salt does not significantly change the number of cations on the surface. The Cl- number density does not show a peak but plateaus indicating no specific interaction of the anions with the CaptoMMC surface.

3 SAM Surface Order Parameters

Figure 2 illustrates how the carboxylate and phenyl ring density distributions along the surface normal were not changed in the presence of different ions for either the Capto Ligand or Nuvia cPrime. Figure 3 illustrates the location of the carboxylate and alkyl vectors within each SAM strand.

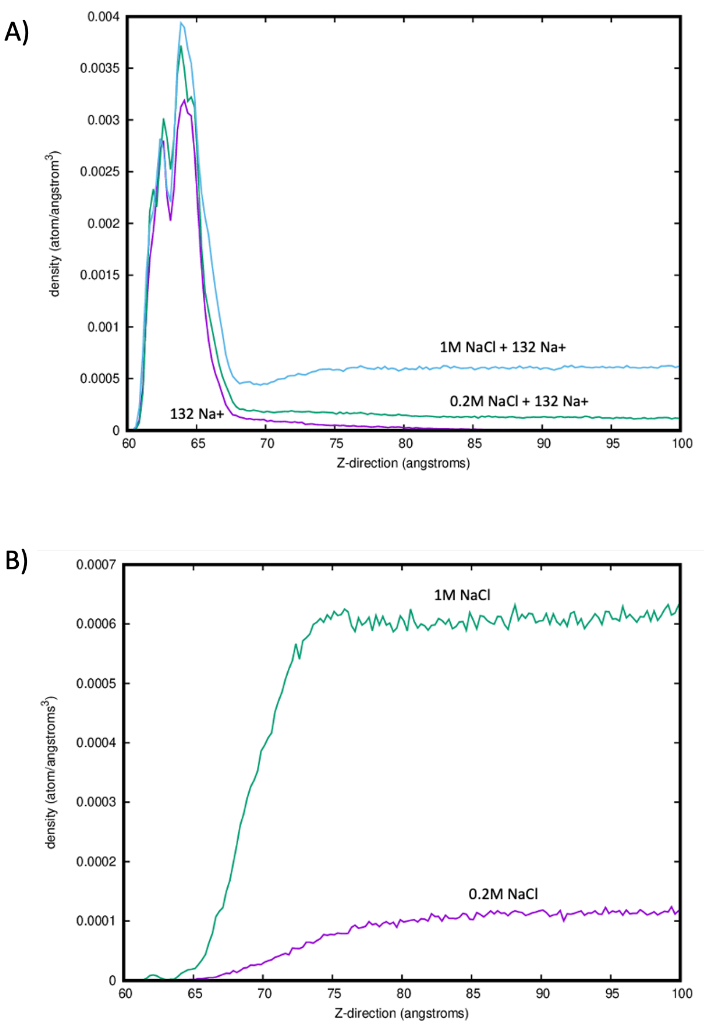

Fig. 1: A) Sodium number density from MD simulations with different background concentrations. B) Chloride number density from MD simulations with different background concentrations.

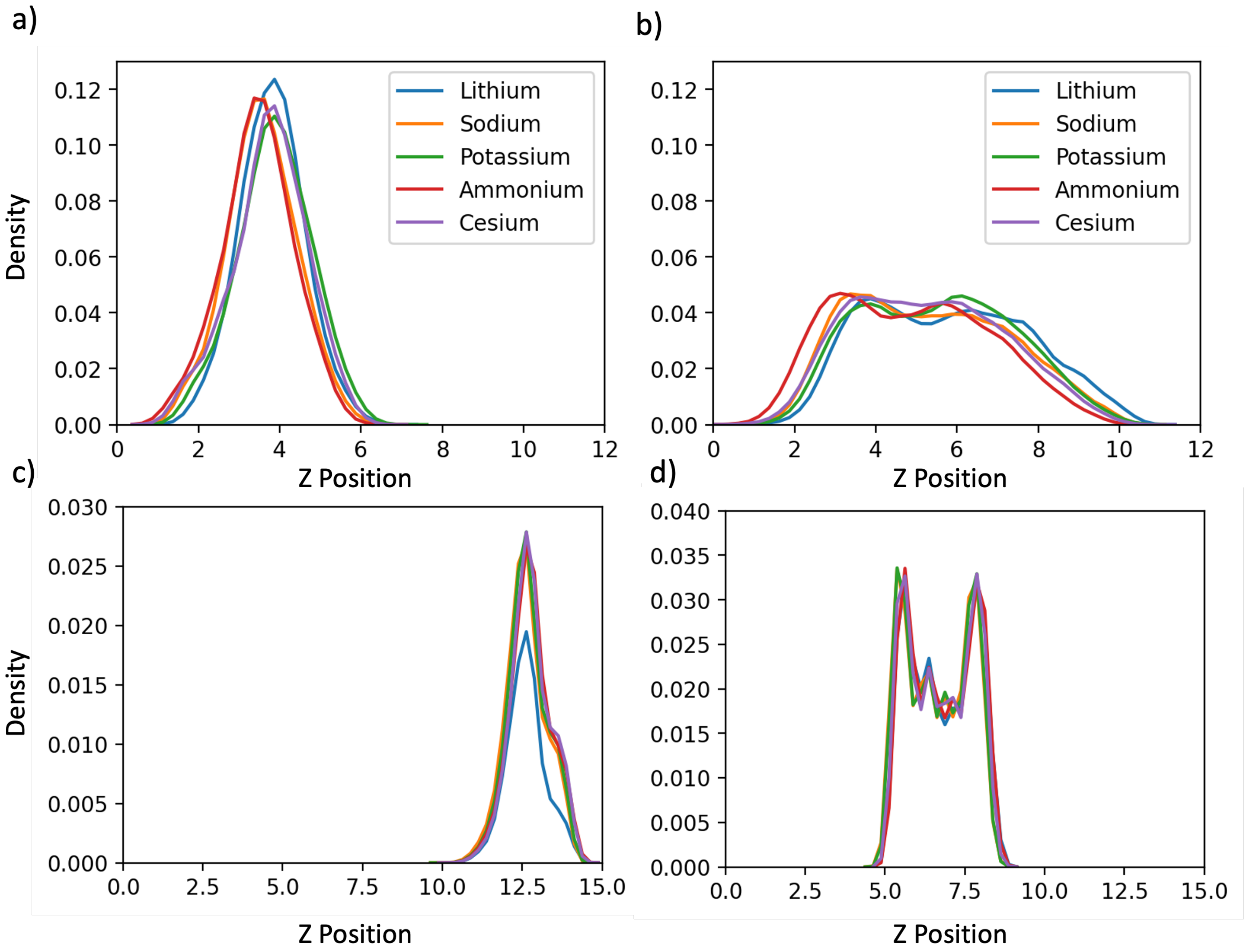

Fig. 2: Density distributions of a) Capto Ligand carboxylate, b) Capto Ligand phenyl ring, c) Nuvia cPrime carboxylate, and d) Nuvia cPrime phenyl ring normal to the plane of the surface in simulations containing different ions. Density are in units of atoms/Å^3.

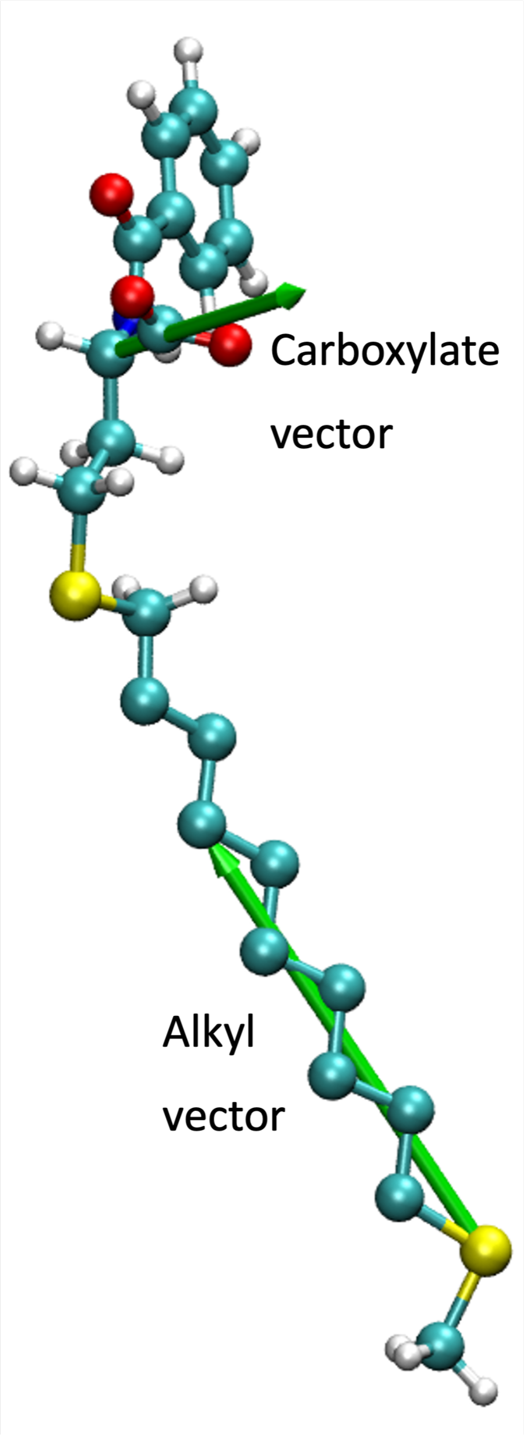

Fig. 3: Illustration of the carboxylate and alkyl vectors.
